# Synthesis and Characterization of Copolymers with Fluorene-di-2-thienyl-2,1,3-benzothiadiazole Units for Application in Optoelectronic Devices

**DOI:** 10.3390/polym17010072

**Published:** 2024-12-30

**Authors:** Elisa Barbosa de Brito, Daniela Corrêa Santos, Taihana Parente de Paula, Andreia de Morais, Jilian Nei de Freitas, Maria de Fátima Vieira Marques, Sergio Neves Monteiro

**Affiliations:** 1 Center for Information Technology Renato Archer, (CTI Renato Archer), Rodovia D. Pedro I, Km 143, 6, Campinas 13069-901, SP, Brazil; 2 Instituto de Macromoleculas Professora Eloisa Mano, IMA, Universidade Federal do Rio de Janeiro, IMA—UFRJ, Av. Horacio Macedo 2030, Rio de Janeiro 21941-598, RJ, Brazil; 3 Military Institute of Engineering—IME, Department of Materials Science, Praça General Tibúrcio, 80, Urca, Rio de Janeiro 22290-270, RJ, Brazil

**Keywords:** synthesis of conjugated polymers, D-A copolymers, electroluminescent polymers, red-emitting copolymer

## Abstract

Conjugated donor–acceptor (D-A) copolymers are widely used in optoelectronic devices due to their influence on the resulting properties. This study focuses on the synthesis and characterization of the conjugated D-A copolymer constructed with fluorene and di-2-thienyl-2,1,3-benzothiadiazole units, resulting in Poly[2,7-(9,9-dioctyl-fluorene)-alt-5,5-(4,7-di(2-thienyl)-2,1,3-benzothiadiazole)] (PFDTBT). The synthesis associated with reaction times of 48 and 24 h, the latter incorporating the phase-transfer catalyst Aliquat 336, was investigated. The modified conditions produced copolymers with higher molar masses (Mw > 20,000 g/mol), improved thermal stability and red emission at 649 nm. Furthermore, the resulting D-A copolymers exhibited uniform morphology with low surface roughness (P2—Ra: 0.77 nm). These improved properties highlight the potential of D-A copolymers based on PFDTBT for various optoelectronic applications, including photovoltaics, light-emitting devices, transistors and biological markers in the form of quantum dots.

## 1. Introduction

Conjugated polymers have aroused interest in the area of organic electronics as such materials have semiconductor, conductive, electrochemical and/or optical properties that can be optimized through synthesis and are also processed in solution, thus allowing their application in lightweight and flexible devices [1,2]. This class of polymers reveals beneficial properties relating to a balance between chemical structure and charge delocalization, thus providing high charge mobility and impressive absorption and emission signals [2,3,4]. Therefore, they can be used in various optoelectronic devices, such as organic photovoltaics (OPVs), organic field-effect transistors (OFETs), photodetectors and organic light-emitting diodes (OLEDs) [5,6,7].

Among the most frequently reported conjugated polymers in the literature, the fluorene homopolymer, particularly poly(dioctylfluorene) (PF), stands out due to its emission in the blue light region. When PF is copolymerized with electron-accepting groups, the resulting copolymers exhibit adjusted bandgaps and modified emission colors, covering other regions of the visible light spectrum [8,9,10,11]. The study of these copolymers is promising, both in the area of light-emitting devices [12,13,14] and photovoltaics [15,16,17], as well as in modern medical technologies for observing biomedical images [18,19,20].

Donor–acceptor (D-A) polymers have emerged as promising candidates for organic electronic devices, as their electronic and optoelectronic properties can be tailored through synthesis, allowing control over the electronic structure and the pendent groups [21]. These materials can be processed in solution, enabling straightforward and versatile fabrication methods. D-A type semiconducting copolymers are designed by alternating electron-rich and electron-deficient monomer units through condensation reactions, resulting in linear polymer structures when symmetric monomer units are utilized [22,23,24,25,26,27,28]. These polymers can be used not only in rigid devices but also in wearable devices, demonstrating their versatility [29].

The choice of donor–acceptor units can influence the main material parameters, such as electronic energy levels, optical bandgaps, crystallinity and polymer morphology. Thus, among the units investigated, the incorporation of 2,1,3-benzothiadiazole (BT) in D-A type copolymers is extensively documented in the literature [30,31]. This acceptor unit is widely used in the development of photoluminescent compounds and significantly improves the electronic properties of the resulting copolymers, since it is a unit capable of producing low-bandgap polymers for thin-film transistor devices, for example [32,33,34,35,36,37]. Units based on thiophene derivatives have excellent fluorescent/emissive properties, as well as presenting stable conjugated structures resulting from the enlargement of the thiophene ring. In addition, they have exceptional properties and a special configuration, revealing superior carrier transfer efficiency and a high degree of structural planarity. [38,39]. Another unit, polythiophene (PT), for example, emits red-orange light. However, its luminescence efficiency in the solid state is low, which limits its use in the area of light emission. In this way, the influence of the coupling of thiophene units to fluorene and benzothiadiazole structures is investigated [40,41].

The conjugation between the di-thienyl (DT) and benzothiadiazole (BT) units enhances stability, promotes a planar structure and improves π conjugation of the BT acceptor unit, thereby enhancing its performance [42,43]. Thus, the introduction of the di-2-thienyl-2,1,3-benzothiadiazole (DTBT) acceptor group into the polymer structure acts as a connection with the donor segment, such as fluorene and BT, resulting in an improved electron density, as well as reducing the severe steric hindrance, obtaining a more planarized structure [44,45]. The DTBT unit exhibits high electron and hole injection performance, as well as excellent transport properties. It emits red light, contributing to the red emission in the copolymer when combined with fluorene (a blue emitter) [42]. The combination of fluorene and di-2-thienyl-2,1,3-benzothiadiazole units results in the PFDTBT copolymer [46,47,48].

This copolymer has been reported in the literature [49,50,51,52,53] for its application in photovoltaic devices, either as an active layer or as an electron-blocking layer in perovskite-based devices. In perovskite devices, PFDTBT not only reduces the energy level mismatch between the anode and the CsPbI_2_Br perovskite but also passivates surface defects of the perovskite [54]. Additionally, it has been utilized in biosensors and imaging devices, such as quantum dot semiconductors (Pdots), highlighting its versatility as a polymeric semiconductor [18,55]. The present work introduces a new aspect by presenting the synthesis of PFDTBT with the addition of the interfacing agent Aliquat 336 in the reaction medium. In this way, we explore how this modification influences the chemical and electronic properties of the copolymer, aiming to expand its potential future applications.

## 2. Experimental Section

### 2.1. Materials

The monomers 9,9-dioctyl-fluorene-2,7-bis(pinacol ester of diboronic acid) and 4,7-Bis(5-bromo-2-thienyl)-2,1,3-benzothiadiazole were purchased from Henan Alfachem Co., Ltd., (Zhengzhou, China); the palladium catalyst ((Pd(PPh_3_)_4_) was supplied by Sigma-Aldrich, (São Paulo, Brazil); and the phase-transfer agent, trioctylmethylammonium chloride (Aliquat 336), was supplied by Oakwood Chemical. The sodium carbonate used was purchased from Sigma-Aldrich, and the toluene solvent was supplied by ProQuimios, (Rio de Janeiro, Brazil) and distilled under sodium.

### 2.2. D-A Copolymer Synthesis

#### 2.2.1. Synthetic Procedures of Copolymers

**Figure 1:**
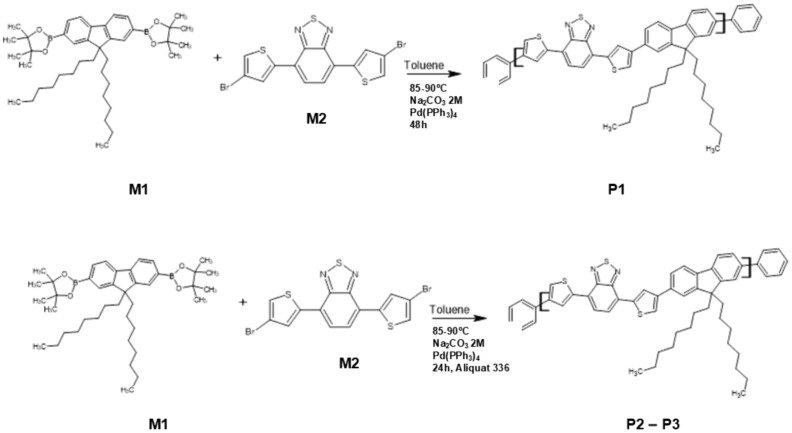
Schematic synthesis of PFDTBT copolymers (P1–P3).

Three samples of poly[2,7-(9,9-di-octyl-fluorene)-alt-4,7-bis(thiophen-2-yl)benzo-2,1,3-thiadiazole] (PFDTBT) were synthesized employing Suzuki–Miyaura coupling polymerization in toluene. The syntheses of PFDTBT copolymers (P1–P3) were conducted following the steps outlined in Figure 1. The copolymerization was carried out according to Suzuki–Miyaura coupling, resulting in materials with conductive properties.

##### Synthesis of PFDTBT Copolymer (48 h, Without Aliquat 336)—P1

The first synthesized D-A copolymer was obtained by adding 1 mmol of 9,9-dioctyl-fluorene-2,7-bis(pinacol ester of diboronic acid) (M1) and 1 mmol of 4,7-Bis(5-bromo-2-thienyl)-2,1,3-benzothiadiazole (M2) together with 0.020 g of Pd(PPh_3_)_4_ in a round-bottom flask. The entire system was weighed in a glovebox under nitrogen. Then, 12 mL of dry toluene and 8 mL of a 2 M potassium carbonate solution were added to this flask. The mixture was stirred vigorously at 85–90 °C for 48 h. After this period, a solution of phenylboronic acid in toluene was added to terminate the polymer chain. The reaction mixture continued to be stirred and heated for an additional 24 h. The crude product was obtained by precipitation in cold methanol after filtration and drying. The red solid acquired was purified by Soxhlet extraction to remove impurities from the catalyst, unreacted monomers and oligomers; the solvent sequence used was acetone, hexane and chloroform. The copolymer collected in the chloroform phase resulted in the PFDTBT sample (P1). ^1^H-NMR spectra of the samples are presented in the Supporting Information (Figure S1).

P1: ^1^H-NMR (δ, ppm) 8.30–6.77 (m, 11H), 2.10–1.99 (m, 3H), 1.65–1.56 (br, 2H), 1.30–0.97 (br, 14H) and 0.85–0.68 (m, 8H).

##### Synthesis of PFDTBT Copolymer (24 h, with Aliquat 336)—P2 and P3

In another round-bottom flask, the same quantities of monomers and catalysts were weighed, as well as the amounts of solvent and aqueous solution used (2 M K_2_CO_3_), with the difference of adding 10 drops of the transfer agent phase Aliquat 336 into the reaction medium. The polymerization was conducted under the same parameters as PFDTBT, resulting in the PFDTBT_M copolymer, which, when precipitated in cold methanol, proved to be insoluble, and its subsequent purification was not possible.

Based on observations from the synthesis of PFDTBT-M, a new synthesis of the copolymer was conducted under the same conditions, except for a reduced reaction time of 24 h. This modification yielded the sample PDTBT-M24 (P2).

D-A copolymers P1 and P2 were then purified by Soxhlet extraction using a sequence of solvents, such as acetone, hexane and chloroform, with the synthesized copolymers collected in the latter. However, a considerable amount of the sample in P2 was not completely soluble in chloroform, requiring submission to the Soxhlet extraction process in chlorobenzene. The copolymer collected in this phase was named PFDTBT-M24CB (P3).

P2: ^1^H-NMR (δ, ppm) 8.19–7.28 (m, 11H), 2.11–1.99 (m, 3H), 1.61–1.43 (br, 2H), 1.23–1.13 (br, 14H) and 0.84–0.60 (m, 8H).

P3: ^1^H-NMR (δ, ppm) 8.24–6.96 (m, 11H), 2.20–1.97 (m, 3H), 1.69–1.51 (br, 2H), 1.22–0.98 (br, 14H) and 0.93–0.64 (m, 8H).

#### 2.2.2. Characterization Methods

To evaluate the properties of the synthesized D-A copolymers, the following instrumentation was used: Prominence^®^ UFLC Shimadzu, (Shimadzu, São Paulo, Brazil) with a Shim-pack GPC-803C column set, 300 × 8.0 mm, Mw = 7 × 10^4^, and PhenogelTM 5 μm Linear (2), 300 × 7.8 mm, Mw = 1 × 10^7^, using CHCl_3_ as an eluent, was used to evaluate molar mass information. A Bruker spectrometer, (Bruker Corporation, Billerica, MA, USA), at 400 and 500 MHz using CDCl_3_ as a solvent was used in order to confirm the synthesized structures. The structure of the copolymers was also investigated by Fourier transform infrared spectroscopy on a PerkinElmer Spectrum 100 (PerkinElmer, São Paulo, Brazil)) infrared spectrometer in ATR mode in the wavelength range 580–4000 cm^−1^. The thermal stability of the samples was evaluated using a TA Instruments Q500 (TA Instruments, New Castle, DE, USA) thermogravimetric analyzer under a nitrogen flow (60 mL/min). The samples, in powder form, were heated from room temperature (RT) to 700 °C at a rate of 10 °C/min. Absorption and emission measurements of samples in solution (1 mg/mL) were carried out on a Shimadzu UV-2550 spectrometer (Shimadzu, São Paulo, Brazil), while samples in solid state (films deposited on glass substrate) were evaluated on an Agilent Cary 60 ultraviolet–visible (UV–vis) spectrometer (Agilent, Santa Clara, CA, USA). Information regarding the absorption and photoluminescence of the D-A copolymer films was obtained using a UV–vis NR Duetta model Horiba fluorescence and absorbance spectrometer. To this end, the films were deposited on a glass substrate by spin-coating at a speed of 3000 rpm for 40 s. Electrochemical measurements (cyclic voltammetry—CV) were conducted in Metrohm Autolab with a three-electrode cell in a 0.1 M solution of tetrabutylammonium hexafluorophosphate (TBAPF6) bubbled with nitrogen in acetonitrile, at a scan rate of 20 mV/s at RT. Ag/AgCl was used as the reference electrode, platinum wire as the counter electrode and polymer-coated platinum as the working electrode. Powder X-ray diffraction (XRD) profiles of the copolymers were obtained using a Rigaku diffractometer model Miniflex (Rigaku, Tokyo, Japan) with a CuK-a radiation source (1.518 Å). The scanning angle was in the range of 2–45°. Finally, to evaluate the morphology of the copolymer films, the Nanosurf C3000 atomic force microscope (AFM) was used, operating in non-contact mode with an area of 10 × 10 μm. The images obtained were processed in Gwyddion software version 2.62 (64 bits).

For the fabrication of the P1–P3 devices, the following processes were performed. The lithographed glass/ITO substrate was cleaned, and subsequently, the PEDOT:PSS (HTL) layer was deposited by spin-coating (3000 rpm, 40 s) and dried on a hot plate at 120 °C for 10 min. The complementary HTL of poly(9-vinylcarbazole) (commercial PVK) was deposited by spin-coating at a speed of 3000 rpm for 40 s. Previously prepared solutions for the P1–P3 copolymers at a concentration of 10 mg/mL were deposited under the complementary HTL with the same parameters used previously. After deposition, the films were annealed on a hot plate at 100 °C for 10 min. Finally, Ca (20 nm) and Al (80 nm) were thermally evaporated at 5 × 10^−6^ mbar inside an MBraun glovebox. After assembly of the PLED devices, they were transferred from the glovebox to a hermetically sealed sample holder for electrical characterization. Current–voltage curves were obtained with a Keithley 2410-C source meter (Tektronix, São Paulo, Brazil). Electroluminescence (EL) spectra were acquired with an Ocean Optics USB2000+ portable fluorimeter (OceanOptics, São Paulo, Brazil) and luminance and CIE coordinates were obtained with a CS-100A color and luminance meter (Konica Minolta–Paramus, NJ, USA). A scanning voltage of up to 28 V was applied to the developed devices.

## 3. Result and Discussion

### 3.1. Molar Mass

**Figure 2:**
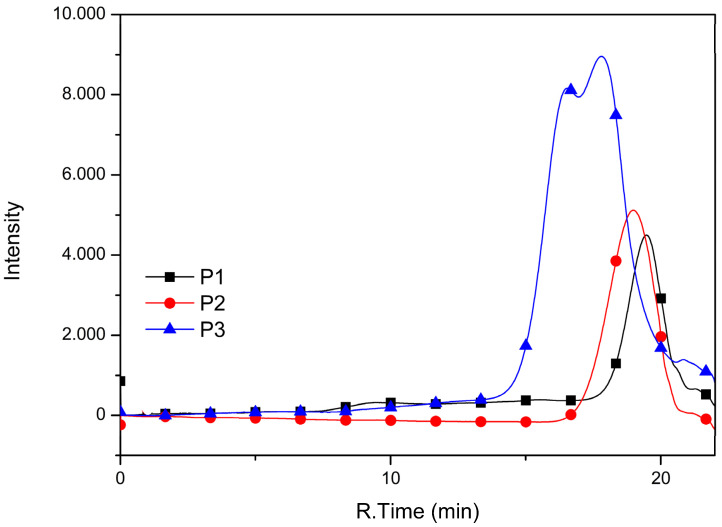
Gel permeation chromatography curves of PFDTBT (P1–P3).

Figure 2 shows the GPC curves of the synthesized copolymers. It is observed that the sample extracted in chlorobenzene (P3) presents greater intensity than the other samples. Table 1 reports data related to these chromatograms.

The weight-average molar mass (M_w_) of the synthesized copolymers ranged from 6700 to 48,900 g/mol, consistent with those reported in the literature [37,38]. However, they exhibited higher dispersity values (Ɖ) ranging from 1.50 to 2.20. For the D-A copolymers synthesized with the addition of Aliquat 336 (P2 and P3), higher molar mass values were observed compared to those synthesized without this agent. Aliquat 336 enhances the solubility and dispersion of reactants, facilitating more efficient interactions between monomers. This leads to a higher degree of polymerization and increased molar mass. This positive effect is particularly evident in Suzuki–Miyaura coupling polymerization, where enhanced phase transfer facilitates improved polymer growth, leading to higher molar masses [56,57].

The data in Table 1 indicate that halving the reaction time did not significantly affect the achieved molar masses or the dispersity.

Zhang et al. [18,41], in their research on biomarker nanoprobes using polymeric quantum dots (Pdots) based on PFDTBT, employed the copolymer supplied by Derthon Optoelectronic Materials Science Technology Co., Ltd. (Shenzhen, China). This copolymer is characterized by an M_w_ > 10,000 g/mol and a Ɖ < 4. Although the authors did not report the reaction conditions, a comparison with the properties obtained in the present syntheses reveals that the molar mass values were higher, and the dispersity slightly exceeded 2, likely due to the polymer being fractionated. The sample synthesized using Aliquat 336 with a reduced reaction time and extracted in chloroform (P2) exhibited a dispersity of 1.75, which is notably lower and falls within the most commonly used fraction. Alghamdi et al. [58] synthesized PFDTBT using palladium acetate (Pd(OAc)_2_) as a catalyst and tri(o-tolyl)phosphine as a phosphine ligand. In their different reaction system, which required the addition of ammonium hydroxide (28%) at the end of the reaction before copolymer precipitation, they achieved an M_n_ of 15,100 g/mol and a dispersity (Ɖ) of 1.57. Additionally, their purification process was more extensive, involving solvents such as methanol, acetone, hexane, toluene and chloroform, with the largest fraction collected in chloroform. Comparing with our data, it is observed that in a shorter and optimized reaction time, the PFDTBT copolymer was acquired with a molar mass very close (P2) to that reported by Alghamdi et al. [58]. In addition, higher molar masses were achieved in the chlorobenzene fraction (P3).

In this way, the synthesis and purification methodology employed in this study demonstrated excellent efficiency in the chaining of fluorene and di-2-thienyl-2,1,3-benzothiadiazole meres, while also achieving lower energy consumption.

### 3.2. Fourier Transform Infrared (FTIR) Spectroscopy

**Figure 3:**
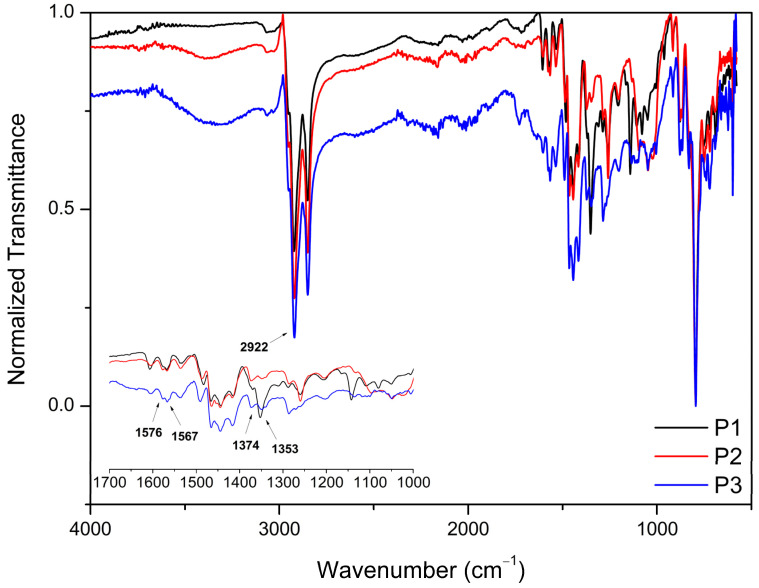
FTIR spectrum of PFDTBT (P1–P3).

Figure 3 shows the FTIR spectra of the synthesized PFDTBT copolymers (P1–P3).

The FTIR spectra of the red copolymers P1–P3 are normalized to the transmission peak of the C(sp^3^)-H stretching vibration at 2922 cm^−1^, and they present the same profile demonstrated in the work of Wu and collaborators [59]. Furthermore, the transmission peaks at 1576, 1567, 1374 and 1353 cm^−1^ (featured) are characteristic transmission bands of 2,1,3-benzothiadiazole (BT) [60].

### 3.3. Thermal Properties and XRD Studies

**Figure 4:**
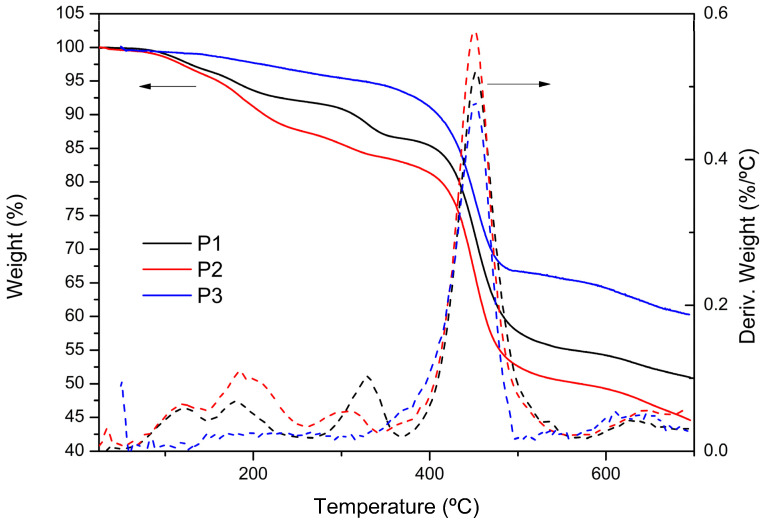
TG/DTG curves of red copolymers (P1–P3).

Figure 4 shows the TG/DTG curves of the red-emitting copolymers. All samples exhibit mass loss at temperatures below 400 °C, which is associated with the chains having lower molar masses. Table 2 presents the data corresponding to these curves.

According to Lee et al. [49], the maximum degradation rate temperature for the PFDTBT copolymer was 428 °C. In contrast, all synthesized samples in this study exhibited T_max_ values exceeding 450 °C, demonstrating improved thermal stability. This enhanced thermal stability is associated with the higher molar masses obtained for the polymers, which reduce the proportion of reactive chain ends and strengthen intermolecular interactions. The use of Aliquat 336 as a phase-transfer catalyst significantly contributed to this improvement by promoting efficient polymerization and yielding polymers with superior structural integrity and thermal resilience.

**Figure 5:**
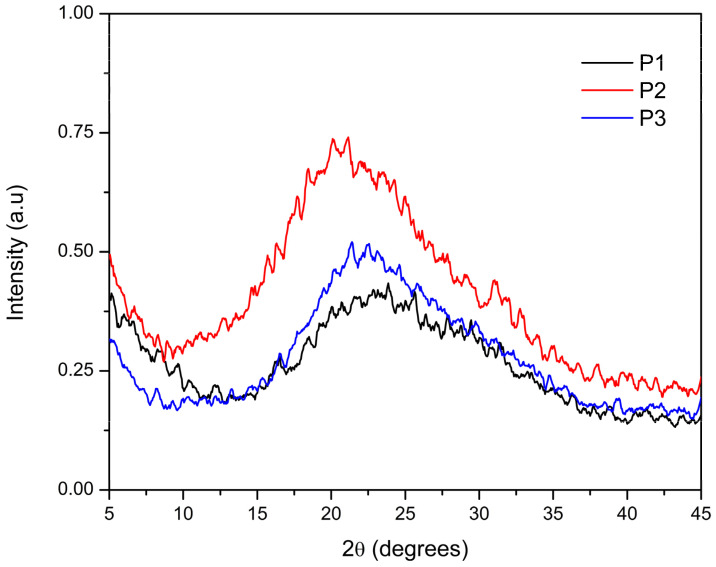
X-ray diffraction analysis of red PFDTBT copolymers.

According to Figure 5, the XRD analysis shows that the P1 sample presented a single, broad diffraction peak at 2θ = 23.1°, indicating a mostly amorphous structure. The sample synthesized with a 24 h reaction time (P2) exhibited a peak at 2θ = 20.4°. Finally, the sample extracted in chlorobenzene (P3) displayed strong diffraction at 2θ = 21.9°. These diffraction patterns correspond to amorphous halos, suggesting the formation of an amorphous polymer with a certain degree of molecular organization through π-π stacking. Liu et al. [61] reported a strong diffraction peak at 2θ = 21.3° for the PFDTBT copolymer and attributed this to the π-π stacking spacing between polymer layers along the z-direction. By contrast, the peak at 2θ = 3.9° was attributed to the spacing between the polymer main chains, separated by the side chains in the plane [61]. Conductive materials based on the aforementioned units are amorphous in nature [44,62,63,64], and a greater crystalline tendency is obtained only if fluorination is performed on the copolymer structure, which provides a limitation to rotation and reinforces coplanarity. Such effects lead to better molecular packing in the solid state [65].

According to Lee et al. [49], fluorene-based polymers are generally amorphous. In the case of the PFDTBT copolymer, no sharp peaks were observed in the XRD patterns other than a broad halo in the range of 15–30°, confirming that the synthesized copolymers predominantly exhibit amorphous characteristics [49,66].

### 3.4. Absorption and Photoluminescence Measurements

**Figure 6:**
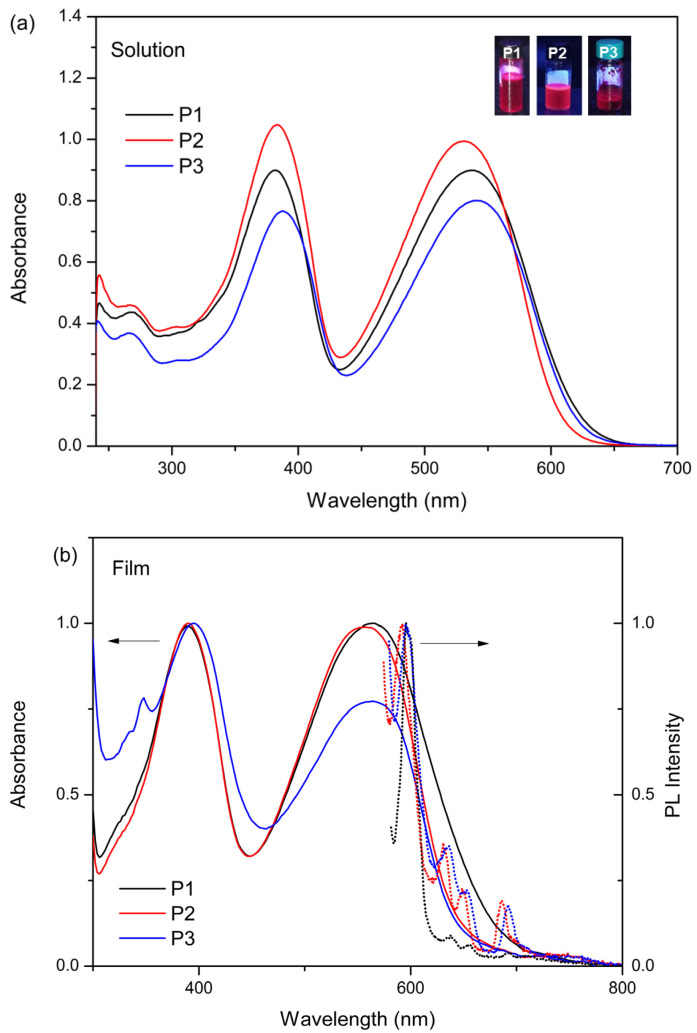
(**a**) UV–vis spectroscopy curves of red copolymers (P1–P3) with their respective emissions in a dark chamber (UV lamp: 365 nm). (**b**) Absorbance and photoluminescence (exc. 560 nm) of P1–P3 films.

Figure 6 shows the absorption spectra of red-light-emitting copolymers based on PFDTBTs. The data indicate differences in the absorbance and PL properties among the three samples (Table 3), reflecting their different synthesis conditions and resulting structural properties. Jiang et al. [52] indicated that the PFDTBT copolymer has a maximum absorption wavelength (λ_max_) of about 380 nm. Liu et al. [61] demonstrated that, in addition to this first absorption, the copolymer showed another λ_max_ peak around 550 nm, which was associated with the strong π-π stacking of the main chain.

Lee et al. [49] revealed that for the synthesized PFDTBT copolymer (M_n_ = 19 × 10^3^ g/mol), the maximum absorptions were 384 and 542 nm. The copolymers synthesized in the present work exhibited both absorptions and are similar to each other. The copolymer synthesized without Aliquat 336 showed maximum absorptions at 382 and 536 nm, while the copolymers P2 and P3, both synthesized in the presence of this phase-transfer agent and with a reaction time of 24 h, showed maximum absorptions of 383 and 530 nm for P2 and 388 and 541 nm for P3. Regarding the bandgaps obtained for the synthesized PFDTBT polymers, it is observed that higher values were obtained for the P2 and P3 copolymers with higher molar masses. In the literature, an E_g_^opt^ of 1.86 eV [67] is reported for the PFDTBT copolymer, while values such as 1.93 and 1.91 eV were achieved for P2 and P3, respectively. The synthesis of these copolymers proved effective as semiconductor copolymers since a variety of bandgaps were produced, with just the modification of the reaction time and the addition of Aliquat 336. Such performances were obtained through the chaining of electron-rich and electron-deficient blocks. It can be used in a variety of devices, such as photovoltaics, transistors, light emitters and/or biological imaging [68].

The increased M_w_ of P3 leads to enhanced conjugation length and better π-π stacking interactions, generally resulting in absorption at longer wavelengths [69,70,71]. However, the optical bandgap for P3 is higher than that of P1, which can be attributed to factors such as the degree of crystallinity and molecular packing. The presence of Aliquat 336 and variations in reaction conditions likely influence the electronic environment of the polymer chains, leading to these differences. Polymerization with Aliquat 336 results in more uniform chain growth and slight variations in the polymer structure. These structural changes can affect the molecular arrangement and stacking interactions within the polymer, thereby influencing the absorption properties observed in the UV–vis spectra. Consequently, P3 exhibits a red-shifted absorption peak while maintaining a higher bandgap, suggesting complex interactions affecting its overall electronic properties.

Wu et al. [72] report an emission wavelength (λ_em_) of 627 nm for PFDTBT copolymer diluted in THF. Zhang et al. [18] reported that when commercial PFDTBT (M_w_ > 10,000 g/mol, Ð < 4) was used in the form of Pdots, they showed optimized luminescence properties and fluorescence efficiency, thus achieving an absorption wavelength of 460 nm, while the emission wavelength was 775 nm. Therefore, it is believed that by using the copolymers synthesized in the present work, the Pdots’ properties would be further improved.

Given the aforementioned results, the standout material is the P2 copolymer dissolved in chloroform. This factor is also associated with luminescence quantum efficiency since it presents the highest quantum yield (QY) value. Photoluminescence quantum yield (PLQY) analysis was performed by examining increasing concentrations of P1–P3 copolymers in chlorobenzene, resulting in six solutions in total. Absorbance data were collected across varying wavelengths. Following the analysis, a graph of integrated fluorescence intensity versus absorbance was plotted, which is expected to yield a straight line.

Thus, the fluorescence spectrum was compared to its integrated intensity, using the same parameter for a reference system with a known quantum yield (QY), such as rhodamine in water. Dyes from the rhodamine family have applications in several areas due to their low cost; high solubility in water, ethanol and methanol; and their high fluorescent emission [73,74,75]. Data regarding PLQY are reported in Figure S2 (Supporting Information) and Table 4.

As shown in Table 4, the QYs obtained for the copolymers P1 (M_n_ ~ 4000 g/mol) and P3 (M_n_ ~ 22,000 g/mol), which represent the lowest and highest molar masses achieved in this study, are lower than the standard rhodamine red dye. Despite P3 having the highest molar mass, P2 exhibited superior QY due to more favorable structural characteristics and electronic properties, such as improved molecular packing, enhanced π-π stacking interactions and a more uniform polymer chain growth. These together optimize the emission properties of P2. The increased molar mass of P3 may have decreased packing efficiency. Higher molar mass can result in longer polymer chains that may not pack as tightly due to increased entanglements and less regular alignment. This can disrupt molecular packing and π-π stacking interactions, potentially leading to reduced electronic properties and lower QYs.

### 3.5. Electrochemical Properties

According to Shin et al. [60], the PFDTBT copolymer has an irreversible reduction process and a reduction potential of −1.54 V. The LUMO energy level of this polymer, as determined by the authors, was calculated to be −3.75 eV. The HOMO energy was estimated from the LUMO energy level obtained by cyclic voltammetry, and the optical bandgap energy (E_g_^opt^) was calculated from the UV–vis absorption spectrum, which was 1.92 eV. This approach was necessary because the polymer’s reduction process was not observed within the potential range of 0 to 2.0 V vs. Fc/Fc^+^. This method of calculating energy levels, which yielded a bandgap of 1.92 eV, is commonly reported in the literature [44,69]. Thus, the energy level data in the present work were also estimated from the onset UV–vis spectrum (Figure S2—Support Information). The cyclic voltammogram and the data obtained are reported in Table 5.

**Figure 7:**
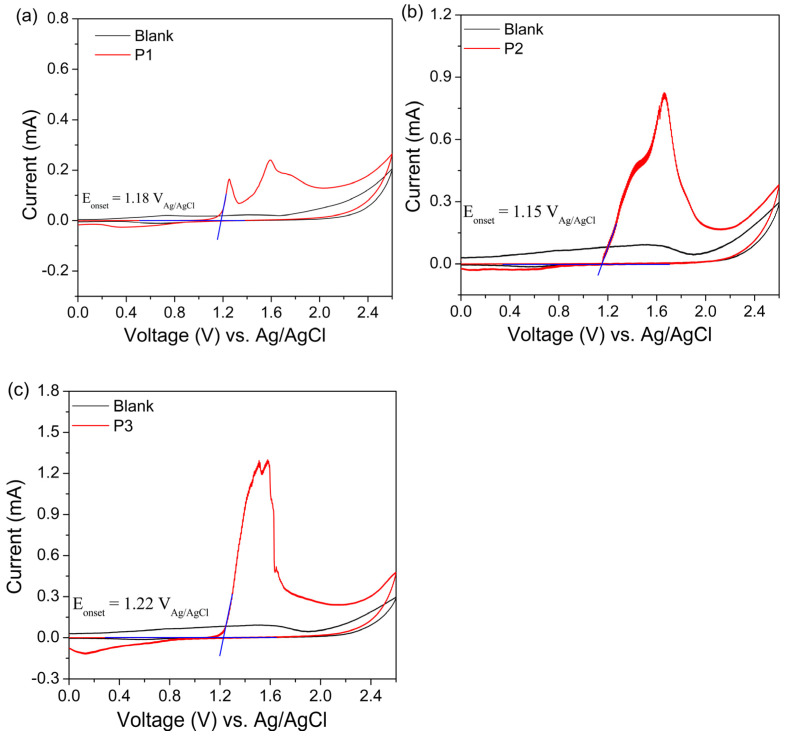
Voltammograms of P1–P3 red copolymers. (**a**) P1 (*y*-axis adjustment—0.1 to 0.8), (**b**) P2 (*y*-axis adjustment—0.1 to 1.2) and (**c**) P3 (*y*-axis adjustment—0.1 to 1.8).

Voltammograms of the synthesized PFDTBT samples are reported in Figure 7, where it is possible to observe the oxidation potentials of 1.18 V for P1, 1.15 V for P2 and 1.22 V for P3. The blue lines shown in the figures below correspond to the tangent of the curves, where they meet is the intersection point (E_onset_).

Lee et al. [49] also report for the PFDTBT copolymer (M_n_ = 19 × 10^3^ g/mol, Ð = 2.6) a bandgap of 1.81 eV, which is very close to the value found for the copolymer synthesized without Aliquat 336 (P1), where E_g_^opt^ = 1.85 eV. It can be noted that the samples synthesized in the presence of this interface agent, which presented higher molar masses, revealed a slight increase in the bandgap. The bandgap of conjugated polymers can be influenced by several factors, such as bond length alternation, resonance energy, planarity of the conjugated structure, intermolecular interactions, π conjugation length and D-A structure [76,77,78]. The use of the interfacial agent in the reaction medium likely promoted greater molar masses, which can enhance the structural organization and increase the rigidity of the polymer chains. These changes can, in turn, affect the planarity of the conjugated structure and the π-conjugation, resulting in slight modifications to the bandgap. Therefore, the alternation of bond lengths in the D-A monomers played a significant role in the energetic separation of the HOMO and LUMO levels in the synthesized copolymers [72,73].

As for the electrochemical properties of the copolymers studied by cyclic voltammetry (CV), as shown in Table 5, the HOMO energy levels were −5.48 eV for P1, −5.45 eV for P2 and −5.52 eV for P3. Lee et al. [49] report that fluorene structures copolymerized with benzochromene moieties (PBCDTBT) have greater electron-donating power than that of fluorene in PFDTBT, and therefore, strongly donating units increase the HOMO energy level. The incorporation of thiophene electron donor units (DTBT) is credited with reducing the HOMO energy levels of the synthesized copolymers, as it can reduce electron donation from the donor segments in the polymer chains, resulting in an expanded bandgap [79].

### 3.6. Morphology of Films

**Figure 8:**
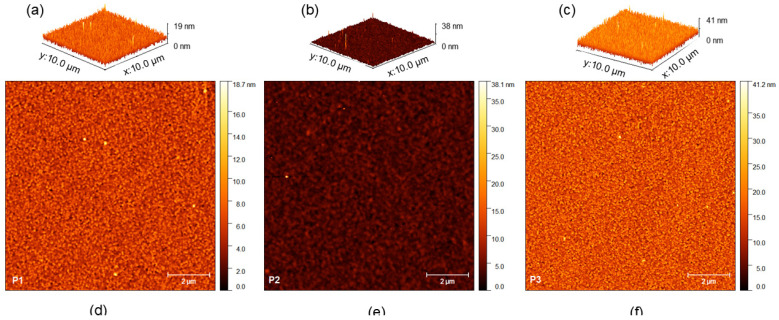
Atomic force microscopy images of P1–P3 copolymer films in chloroform; (**a**–**c**) 3D images of the films (thickness) and (**d**–**f**) images of the appearance of the films (roughness).

Figure 8 shows atomic force microscopy images of P1–P3 copolymers solubilized in chloroform (10 mg/mL). Through the interaction of the cantilever with the sample, it was possible to evaluate the thickness of each film produced. Among the samples, it is clear that the one with the smallest thickness is sample P1, which refers to the material produced without the action of Aliquat 336.

According to the data contained in Table 6, the lowest roughness, whether average (Ra) or root mean square (RMS), is associated with P2, the copolymer with the highest PLQY among the samples, which has M_n_ > 12,000 g/mol and Ɖ = 1.75.

When applied in photovoltaic systems, a lower roughness of the polymer film favors the dissociation of the exciton and the charge transport [80,81].

The greatest thickness was credited to the copolymers P2 and P3. In the literature, it is reported that a polymer thickness of less than 40 nm reveals a tendency to reduce the external quantum efficiency (EQE) across the entire absorption spectrum [82]. Therefore, the data obtained for the studied copolymers represent a promising feature for future device development. Among the copolymers, P2 exhibited a better balance of properties when considering the relationship between thickness and roughness. Roughness is a critical factor for both the successful deposition of subsequent layers in devices and the efficient transfer of charges between them, highlighting the potential of P2 for improved device performance.

### 3.7. Application in Light-Emitting Devices

**Figure 9:**
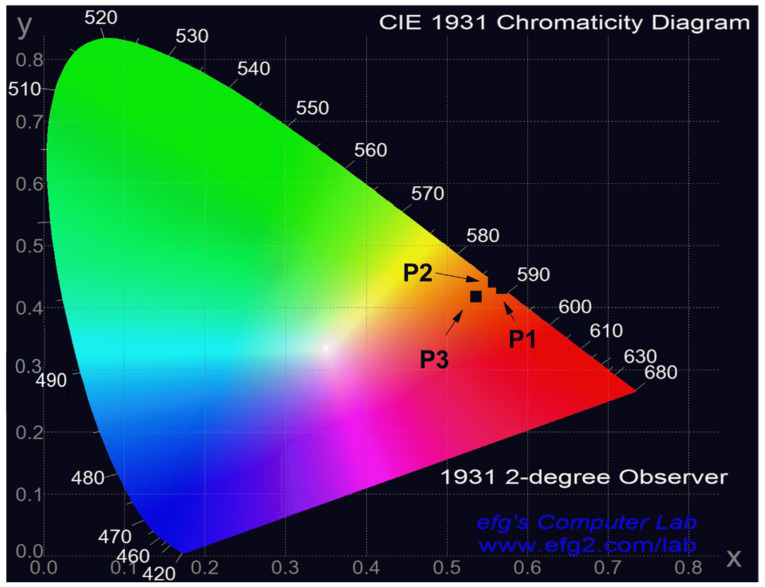
CIE coordinate of P1–P3-based devices.

To evaluate their potential application in light-emitting devices, the synthesized red copolymers were incorporated as the emitting layer in a device structure consisting of glass/ITO/PEDOT:PSS/PVK/P1-P3/Ca/Al. The chromaticity coordinates of the devices constructed with the respective copolymers are presented in Figure 9.

The electroluminescent data obtained for the devices are summarized in Table 7, with additional information provided in the Supplementary Material (Figures S4 and S5). These figures show that the P2 copolymer exhibits higher luminance compared to the other devices, which can be attributed to its superior photoluminescence quantum yield (PLQY).

Given the high drive voltages, future optimizations are needed to increase the emission efficiency, such as doping the copolymers, as well as a study of the other components of the device, to better adjust the energy levels [83,84,85,86]. However, the results indicate that these materials, particularly the P2 copolymer, have potential for application in display and/or lighting devices, provided that the device construction conditions are further refined.

## 4. Conclusions

In this study, three PFDTBT copolymers were synthesized by varying the reaction time from 48 to 24 h and incorporating the interfacing agent Aliquat 336 into the polymerization medium. These modifications significantly influenced the molar mass, with copolymer P2 reaching Mn > 12,000 g/mol and P3 reaching Mn > 22,000 g/mol. All synthesized polymers demonstrated excellent thermal stability, with T_max_ values exceeding 450 °C, indicating their robustness for high-performance applications. The emissive properties of the copolymers revealed that P2 exhibited a higher photoluminescence quantum yield than the other samples and the rhodamine standard in water. Photoluminescence tests confirmed the red light emission of the synthesized copolymers, a key feature for optoelectronic applications. Furthermore, the films produced from these materials showed low surface roughness, suggesting efficient charge carrier transport without the formation of trap regions. These characteristics increase the suitability of the material for electronic and photonic devices. Furthermore, the synthesized copolymers demonstrated an optimized bandgap, making them highly compatible with various material mixtures in photovoltaic applications. These results highlight the significant potential of the synthesized materials for use in optoelectronic devices, such as displays, lighting systems and photovoltaic cells. An initial study was developed regarding the use of the synthesized copolymers as an emitting layer, and these revealed the emission of red light. Among the samples, P2 stands out, since it reveals greater luminance than the other copolymers, as well as greater current efficiency, demonstrating its applicability for the lighting and display area. Future optimizations are necessary to further enhance potential. Thus, it is clear that the combination of customized synthesis strategies, high thermal stability, excellent emissive properties and promising electrical characteristics highlights the versatility and applicability of these materials in advanced technological applications.

## Figures and Tables

**Table 1:** GPC data of P1–P3 copolymers.

Copolymer	Reaction Time (h)	Yield (%)	M_n_ (g/mol)	M_w_ (g/mol)	Ɖ
P1	48	76	4353	6690	1.53
P2 (+Aliquat) *	24	67	12,480	20,156	1.75
P3 (+Aliquat) **	24	20	22,524	48,952	2.17

* P2 = CF fraction; ** P3 = CB fraction.

**Table 2:** Results of thermostability and glass transition temperature of polymers P1–P3.

Copolymer	TGA			
	T_10%_ (°C)	T_onset_ (°C)	T_max_ (°C)	Residue (%)
P1	314.8	428.3	452.1	5.1
P2	213.2	428.2	450.3	4.4
P3	409.3	413.1	450.9	6.0

**Table 3:** Photophysical properties of P1–P3 red copolymers.

Sample	λ_máx_^sol^ (nm)	λ_máx_^film^ (nm)	λ_onset_ (nm)	PL^560^ (nm)	E_g_^opt^ (eV) *
P1	382, 536	390, 564	669	595	1.85
P2	383, 530	392, 554	642	597	1.93
P3	388, 541	396, 564	648	597	1.91

* Estimated from the absorption onset collected from the UV–vis spectra of the films. E_g_^opt^ = 1240/*λ* (nm).

**Table 4:** Data relating to PLQY analysis of P1–P3 copolymers.

Sample	Angular Coefficient	Quantum Yield (%)
Rhodamine	182.47	0.31 (standard)
P1	71.89	0.15
P2	250.57	0.55
P3	124.08	0.27

**Table 5:** Electrochemical properties of P1–P3 copolymers.

Copolymer	E_HOMO_ (eV)	E_LUMO_ (eV)
P1	−5.48	−3.63
P2	−5.45	−3.52
P3	−5.52	−3.61

Estimated from cyclic voltammetry. *E*_HOMO_ = −(4.8 + *E*_onset_ ^ox^) [eV]; *E*_LUMO_ = E_HOMO_ + E_g_^opt^ [eV]. E_g_ = 1240/λ (nm).

**Table 6:** Data obtained from PFDTBT films (P1–P3) through atomic force microscopy analysis.

Sample	Average Roughness (Ra) (nm)	Root Mean Square Roughness (RMS) (nm)	Thickness (nm)
P1	1.11	1.40	18.7
P2	0.77	0.99	38.1
P3	2.94	3.68	41.2

**Table 7:** Characteristics of PLEDs containing red copolymers P1–P3 as the emitting layer.

EL Layer	V_on_ (V)	L_max_ (cd/m^2^)	η_curr_ (mcd/A)	CIE (x, y)
P1	13	26	11	(0.55; 0.43)
P2	18	116	49	(0.54; 0.44)
P3	16	93	16	(0.52; 0.41)

## Data Availability

The original contributions presented in the study are included in the article/Supplementary Material, further inquiries can be directed to the corresponding author.

## Supplementary Materials

The following supporting information can be downloaded at: https://www.mdpi.com/article/10.3390/polym17010072/s1. Figure S1: ^1^H-NMR spectra of P1–P3 copolymers. Figure S2: PLQY analysis of P1–P3 copolymers and their respective calibration curves. Figure S3: UV–vis absorption spectra (λ_onset_) of P1–P3 copolymers for Eg^opt^ calculation. Figure S4: EL intensity of P1–P3 red copolymers. Figure S5: Current density vs. voltage vs. luminance of P1–P3 red copolymers.
