# Supplementary material for: Synthesis and Characterization of Copolymers with Fluorene-di-2-thienyl-2,1,3-benzothiadiazole Units for Application in Optoelectronic Devices
Source: Polymers (Basel). 2024 Dec 30;17(1):72. doi: 10.3390/polym17010072 (PMC11722960; doi:10.3390/polym17010072)
Supplement: Supplementary file 1 [file polymers-17-00072-s001.zip › polymers-3272789-supplementary.pdf]

Synthesis and Characterization of Copolymers with Fluorene-di-2-thienyl-2,1,3-benzothiadiazole Units for Application in Optoelectronic Devices

Elisa Barbosa de Brito [1,2,*], Daniela Corrêa Santos [2], Taihana Parente de Paula [2], Andreia de Morais [1],
Jilian Nei de Freitas [1], Maria de Fátima Vieira Marques [2] and Sergio Neves Monteiro [3]

1 Center for Information Technology Renato Archer, (CTI Renato Archer), Rodovia D. Pedro I, Km 143, 6, Campinas 13069-901, SP, Brazil;
2 Instituto de Macromoleculas Professora Eloisa Mano, IMA, Universidade Federal do Rio de Janeiro, IMA—UFRJ, Av. Horacio Macedo 2030, Rio de Janeiro 21941-598, RJ, Brazil;
3 Military Institute of Engineering—IME, Department of Materials Science, Praça General Tibúrcio, 80, Urca, Rio de Janeiro 22290-270, RJ, Brazil;
* Correspondence: elisabarbosabrito@gmail.com

Support Information

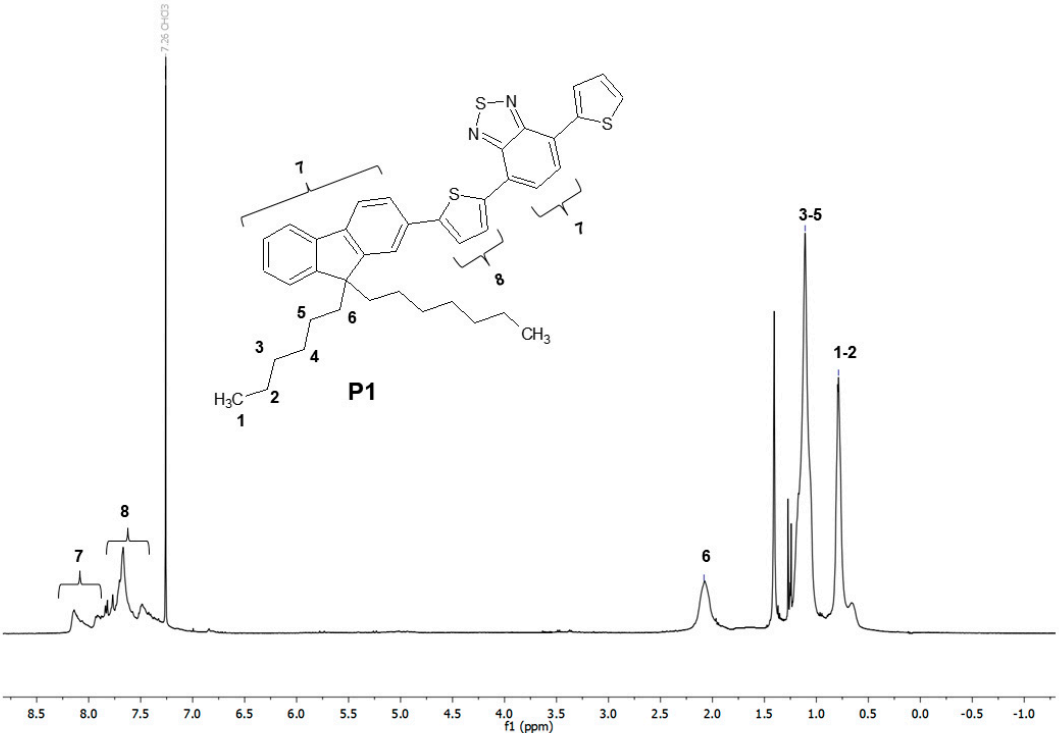

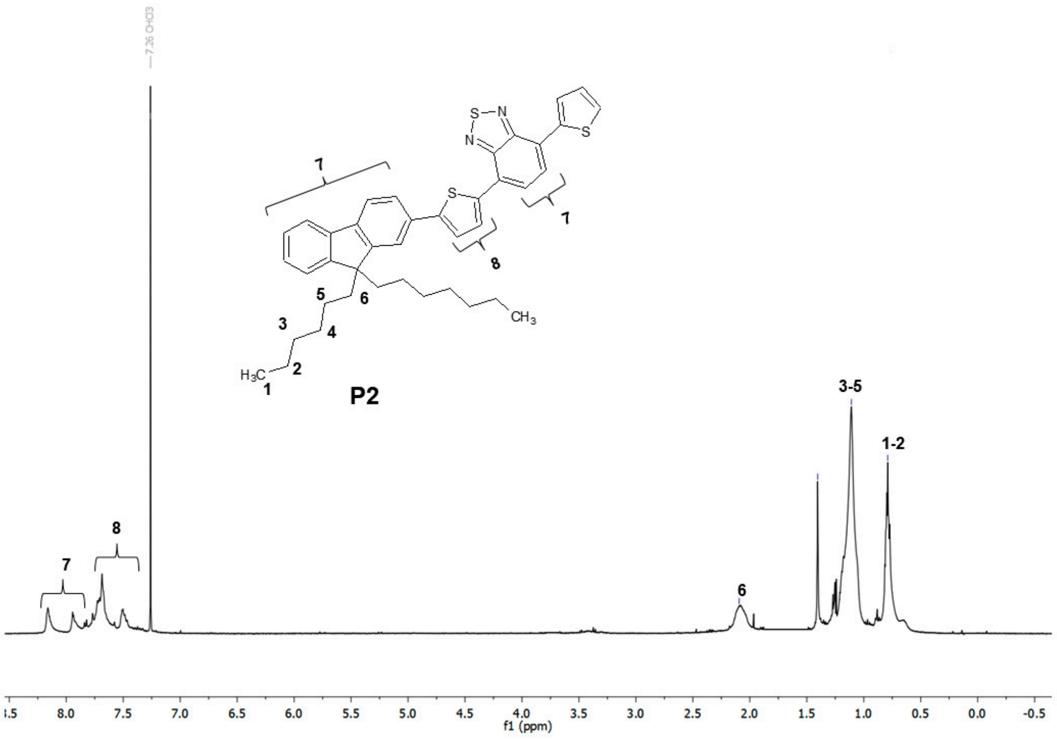

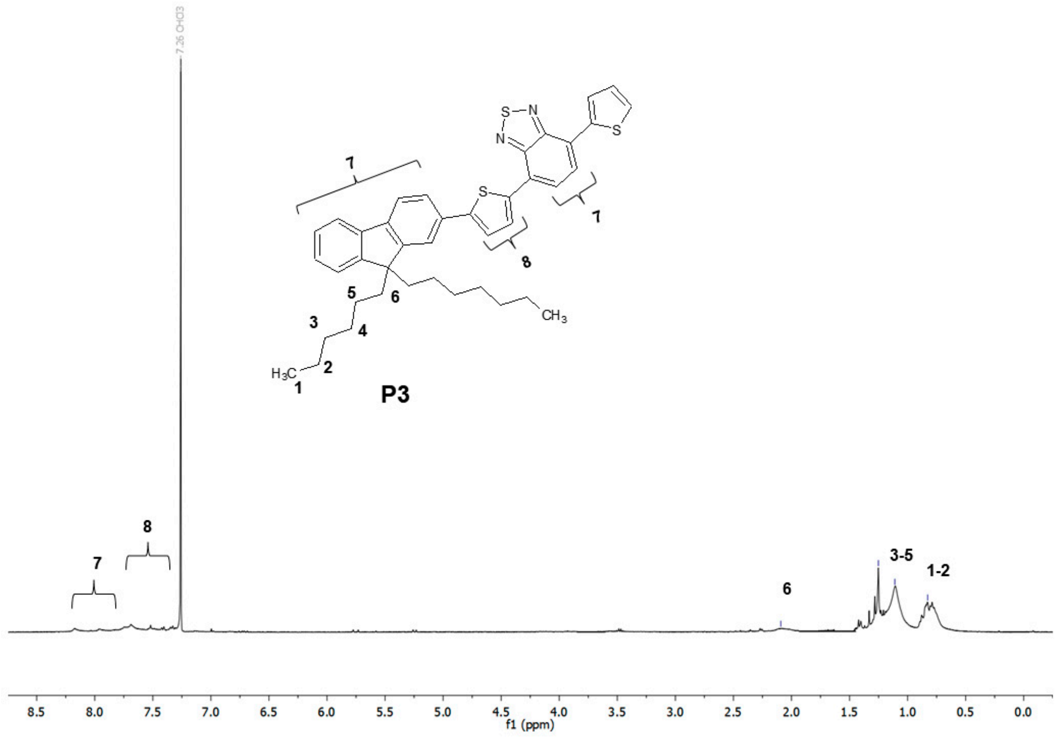

Figure S1: ^{1}H-NMR spectra of P1-P3 copolymers.

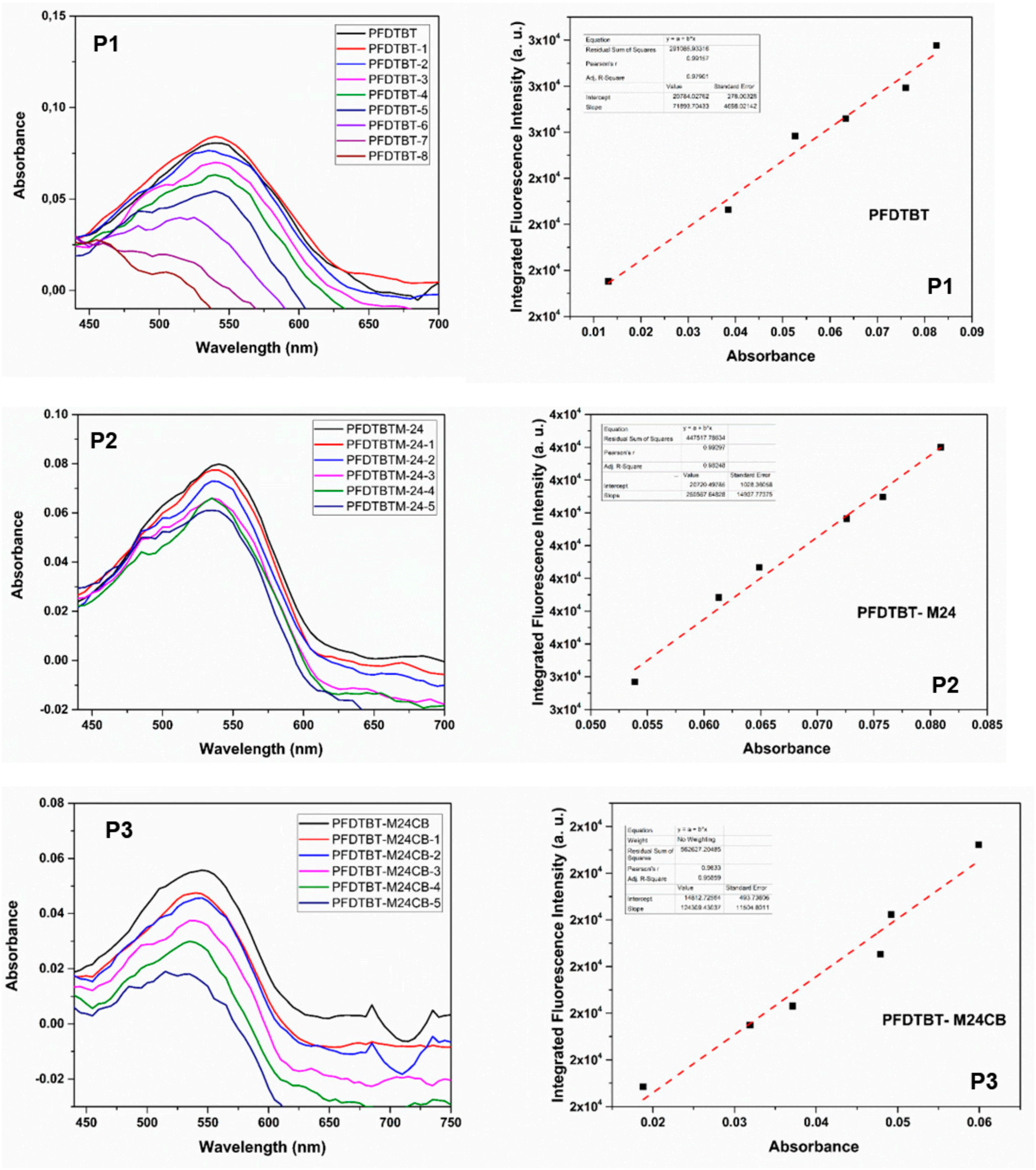

Figure S2: PLQY analysis of P1-P3 copolymers and their respective calibration curves

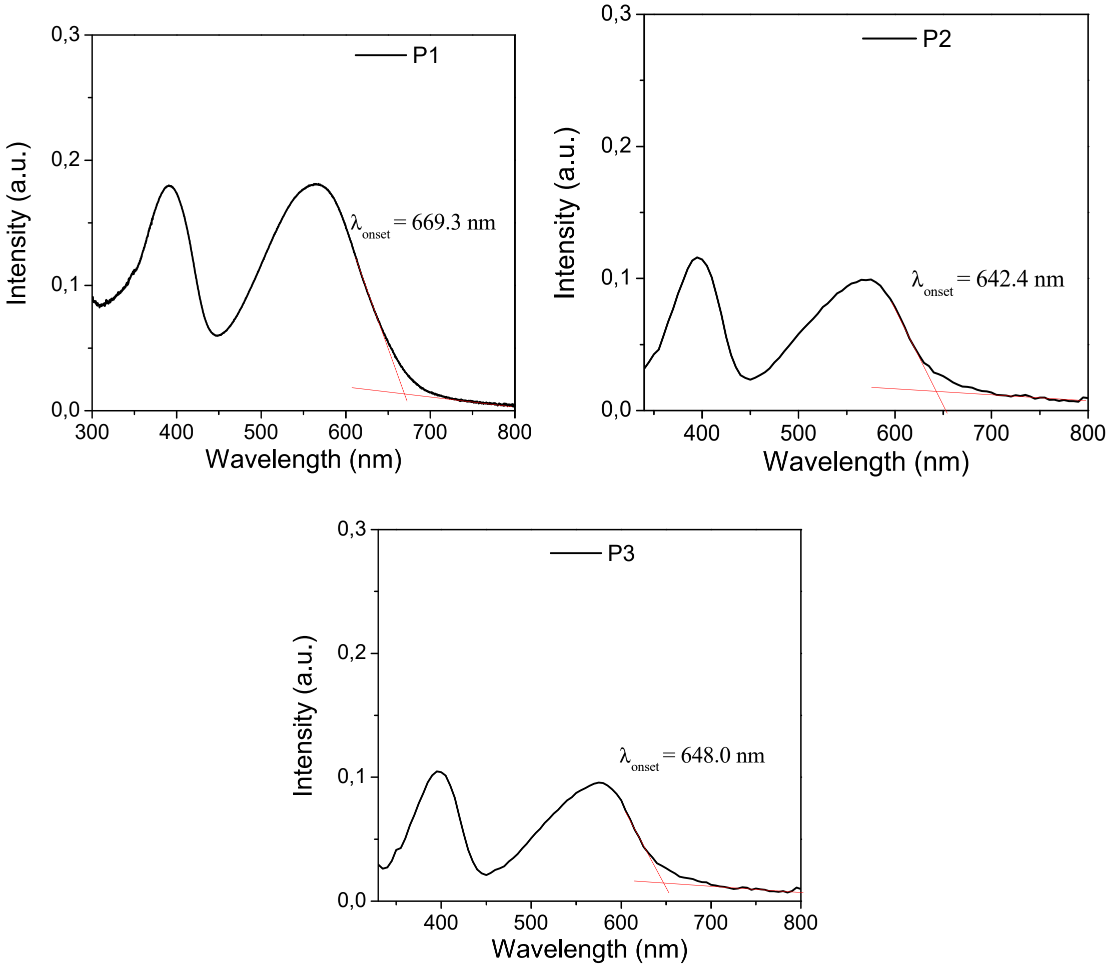

Figure S3: UV-vis absorption spectra - λ_{onset} of P1-P3 copolymers for Eg^{opt} calculation.

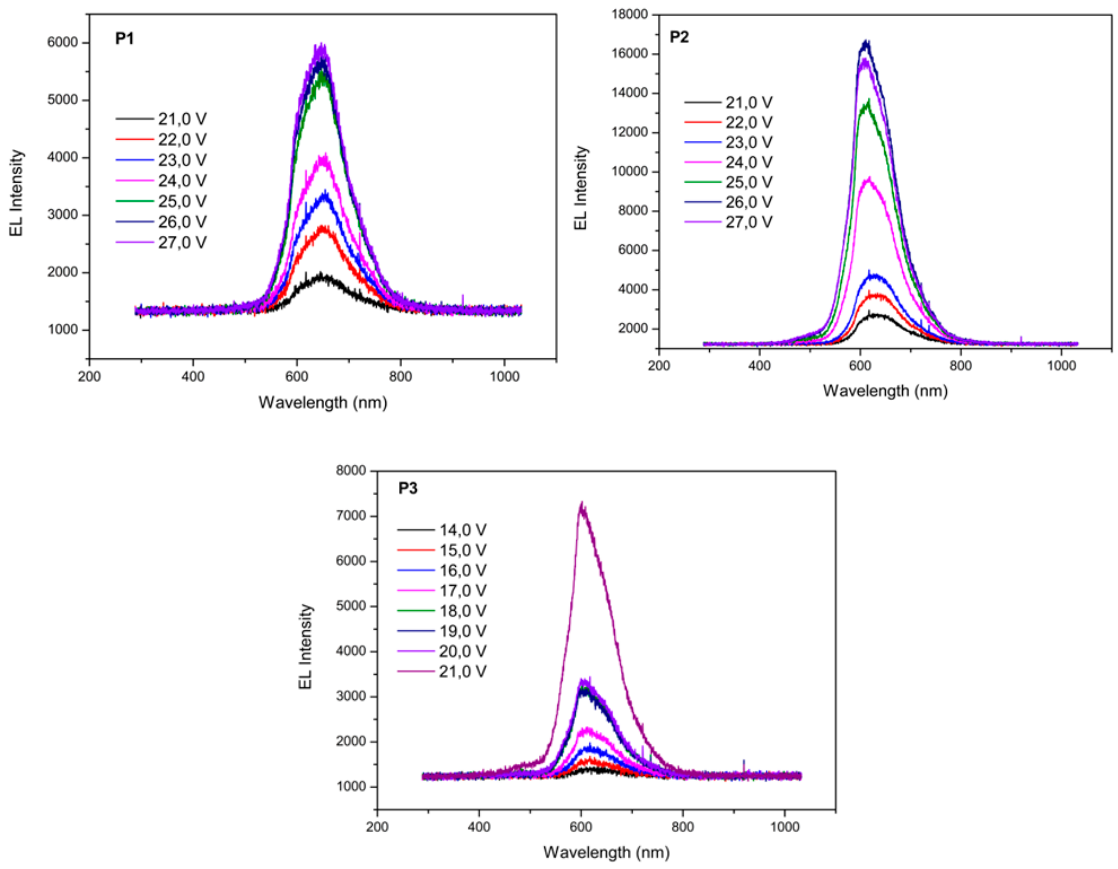

Figure S4: EL Intensity of P1-P3 red copolymers.

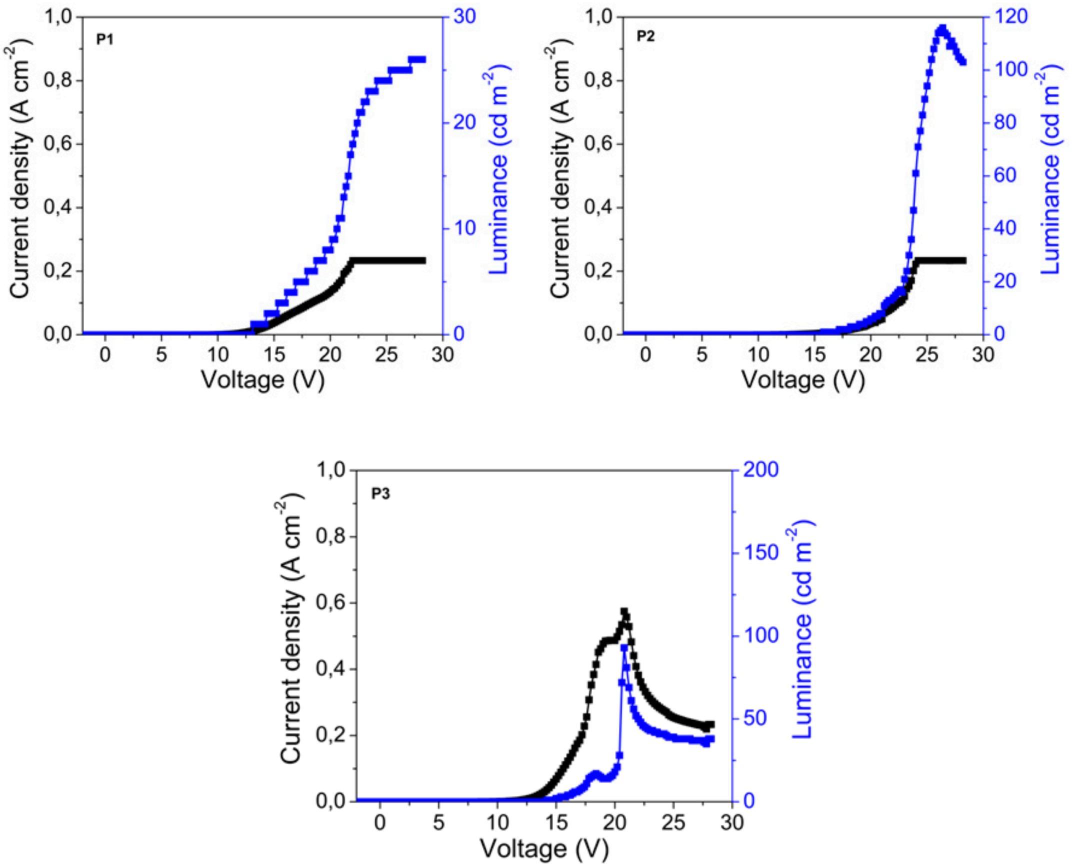

Figure S5: Current density vs voltage vs luminance of P1-P3 red copolymers.
